# Predictive validity on clinical item-level of the HKT-R divided into clinical patient classes

**DOI:** 10.1186/s12888-023-04994-4

**Published:** 2023-07-12

**Authors:** Iris Frowijn, Erik Masthoff, Stefan Bogaerts

**Affiliations:** 1 Department of Developmental Psychology, Tilburg University, Tilburg, the Netherlands; 2 Fivoor Science and Treatment Innovation (FARID), Rotterdam, the Netherlands

**Keywords:** Risk assessment, Latent class analysis, HKT-R clinical risk factors, Predictive validity, Violent recidivism

## Abstract

**Background:**

Because of the heterogeneity of forensic groups, latent class analysis (LCA) can allow for the formation of stronger homogeneous patient classes, which can improve the predictive validity of forensic risk assessment tools, such as the Historical Clinical Future – Revised (HKT-R), which was used in this study. In particular, dynamic clinical risk and protective items are important in treatment and are obligatory assessed annually for every forensic patient with a TBS measure in the Netherlands. Therefore, this study investigated the predictive validity of the HKT-R at clinical item-level per patient class.

**Method:**

A cohort of 332 forensic patients, who were discharged from highly secured Forensic Psychiatric Centers/Clinics (FPCs) in the Netherlands between 2004 and 2008, was followed. LCA was performed to cluster this group of patients based on psychopathology and criminal offenses. The predictive validity of the HKT-R clinical items by class was assessed with official reconviction data two and five years after discharge as outcome measure.

**Results:**

Four classes were identified. The predictive validity of the HKT-R clinical items showed differences between and within classes on admission or discharge, and for predicting violent reoffending after two or five years after discharge.

**Discussion:**

Different risk/protective factors of the HKT-R may play a role for different subgroups of patients. Therefore, this heterogeneity should be considered for any measure or intervention.

## Introduction

When individuals commit crimes, the criminal justice system aims to reduce the risk of violent recidivism in order to protect society, by, for instance, sentencing them to imprisonment and/or forensic treatment within a legal framework [1]. In the Netherlands, the imposition of a TBS measure (Entrustment Act; [1]) is an example of the latter. The treatment offered in this context to forensic psychiatric patients in high-security clinics, is then based on the principles of the Risk-Need-Responsivity model (RNR; [2, 3]). According to this model, forensic treatment is primarily driven by an offender’s unique pattern of offense-related risk and protective factors, captured in the criminogenic needs. Criminogenic needs are the factors linked to recidivism and can be summarized as the Central Eight, which can be divided into the Big Four, which are the most strongly related to recidivism (i.e., criminal history, antisocial cognition, antisocial personality patterns, and antisocial associates), and the Moderate Four that are less strongly and more indirectly related to recidivism (i.e., problems at school/work, family/marital problems, substance abuse, and lack of prosocial leisure/recreation) [3–5]. In order to keep track of these factors during treatment, in the Netherlands, risk assessment must be performed periodically and at least once a year [6].

An incorrect risk assessment can have serious consequences both for the patient in case of overrating (unnecessary prolongment of treatment), and society in case of underrating (irresponsible/high-risk release), which is why sufficiently reliable and valid measurements are required [7]. To support and standardize this procedure, risk assessment instruments with Structured Professional Judgment (SPJ) procedures are preferred over clinical assessment and are used in most developed countries [8]. In the Netherlands, the *Historisch Klinisch Toekomst-Revised* (Historical Clinical Future-Revised; HKT-R; 9) is mainly used, which is comparable to the Historical Clinical Risk-management-20 Version 3 (HCR-20 ^V3^; [10]). The HKT-R is an SPJ risk assessment instrument to assess the risk on violent recidivism [8]. It consists of three domains: the historical domain with only static risk factors of the patients’ (criminal) background, the clinical domain containing dynamic risk and protective factors assessed on past 12-month behavior, and the future domain with potential risk factors if a patient would leave the facility [8].

The HKT-R has a modest predictive validity for violent reoffending for two years (AUC = 0.78) and marginal for five years follow-up of time at risk (AUC = 0.68) [11]. The HKT-R clinical items play an important role to support decisions on, for example, leave modalities, discharge procedures or prolongation of the TBS measure [9, 12]. In fact, the predictive validity for violent recidivism of the clinical domain of the HKT-R showed to be marginally predictive within two (admission: AUC = 0.62; discharge: AUC = 0.63) as well as marginally predictive within five years of time at risk (admission: AUC = 0.69; discharge: AUC = 0.62) [11]. More recently, using a network approach, it was found that there are individual differences in associations between dynamic risk and protective factors at item-level (rescaled; see Table 1) [13]. Though, the dynamic risk and protective factors are not yet linked to violent recidivism at the item-level, while not all items may be equally strongly associated to violent recidivism.

**Table 1:** Items of the HKT-R

Historical items	Clinical items	Future items
H01 Legal history	*Risk factors*	T01 Agreement on future conditions
H02 Violation of terms	K02 Psychotic symptoms	T02 Accommodation
H03 Age at first conviction	K03 Addiction	T03 Financial situation
H04 Type of victims	K04 Impulsivity	T04 Employment
H05 Network influence	K05 Antisocial behavior	T05 Daily activities
H06 Behavioral problems before the age of 12	K06 Hostility	T06 Social network
H07 Victim of violence in youth	K12 Violation of terms and agreements	T07 Stressing circumstances
H08 Treatment history	K14 Influence of risky network-members	
H09 Work history	*Protective factors*	
H10 Past substance use	K01 Problem insight	
H11 History of homelessness	K07 Social skills	
H12 Financial problems	K08 Self-reliance	
	K09 Cooperation with treatment	
	K10 Responsibility for the offense	
	K11 Coping skills	
	K13 Labor skills	

*Note.* Overview of the HKT-R items [9], with the Clinical items subdivided in risk and protective factors according to Bogaerts et al. [13]

Moreover, a high AUC value indicating adequate predictive validity does not necessarily mean that the instrument predicts well for all forensic patients within that population [14]. Because of the strong heterogeneity of the forensic population in terms of psychopathology and offense type [15], it may be considered to identify more homogeneous independent patient classes before investigating the predictive validity of the clinical items of the HKT-R. To do so, several suggestions are made for specific patient classes, mostly based on Latent Class Analysis (LCA; [16]). For instance, in the study by Van Nieuwenhuizen et al. [15], using LCA based on psychopathology and type of criminal offense in a representative sample of TBS patients, five classes of patients were found, visualized in Table 2 *(the psychotic patient with multiple problems; the antisocial patient; the psychotic patient; the patient with sexual problems and sexual crimes; the patient suffering from addiction*; 15). Van der Veeken et al. [17] found four classes (in a similar sample of forensic patients) performing LCA based on the risk factors of the HKT-30 (which is the predecessor of the HKT-R), of which three classes corresponded to aforementioned classes 1 (*the psychotic patient with multiple problems*), 3 (*the psychotic patient)*, and 4 (*the patient with sexual problems and sexual crimes*) [15], though they were named differently. The fourth class was *the antisocial class*, and is described as slightly different from the similarly named class of Van Nieuwenhuizen et al. [15] in type of offense [17].

**Table 2:** Classification of forensic patients based on van Nieuwenhuizen et al. [15] with hypothesized predictive clinical factors

Class	Psychopathology	Type of offense	Hypothesized clinical factors predictive of violent reoffending
1. The psychotic patient with multiple problems	Schizophrenia (or another psychotic disorder) and personality disorder cluster B (or NOS)	Generalist	K01, K04, K05, K06, K09, K11
2. The antisocial patient	Personality disorder cluster B and possibly substance use disorder	Generalist	K03, K04, K05
3. The psychotic patient	Schizophrenia or another psychotic disorder	Specialist	K01, K02, K04, K05, K07, K08, K11
4. The patient with sexual problems and sexual crimes^a^	Sexual-/gender identity disorder	Specialist	K01, K07, K10, K11
5. The patient suffering from addiction	Substance use disorder and a personality disorder NOS	Generalist	K03, K12

*Note.* Classes of patients based on LCA. Psychopathology is based on the DSM-IV-TR [26]. Type of offense is classified in either generalist or specialist, where specialist indicates a specific type (e.g., murder) and generalist indicates multiple types of offenses [15]

^a^Since the HKT-R is not validated for predicting sexual recidivism this cluster should be taken with caution [9]

The classes of Van der Veeken et al. [17] and their risk factors combined with the classes of Van Nieuwenhuizen et al. [15], yield the following characteristics. The class of *psychotic patients with multiple problems* (*mixed profile with multiple problems* in Van der Veeken et al.; [17]) is characterized by hostility and limitations in empathy, emotional reactivity, problem awareness, and coping skills. In addition, treatment responsivity is thought to be problematic in this class, meaning that treatment might take longer compared to the other classes [17]. Next, *the antisocial patient* (*antisocial class* in Van der Veeken et al.; 17) has relatively high impulsivity compared to the other classes and often deals with severe problems related to addiction and antisocial behavior [15]. Furthermore, the class of *psychotic patients* (*psychotic first offender* in Van der Veeken et al.; 17) is characterized by issues with problem awareness and empathy. An additional focus is recommended to be on psychotic symptoms, social skills, and self-reliance skills [17]. More specifically, this group often has neurocognitive impairments in the prefrontal brain area, which may be related to enhanced aggression, impulsivity, and deficits in coping [14]. Likewise, the class of *patients with sexual problems and sexual offenses* (*maladaptive affective disordered profile* in Van der Veeken et al.; 17) represents relatively to the other classes more problems with coping, problem awareness, social skills, and taking responsibility for the crime. A lack of social support is also mentioned as a possible risk in this class [17]. Finally, the fifth class [15], *the patient suffering from addiction*, was more difficult to characterize because it was not found by Van der Veeken et al. [17]. Yet, in terms of risk factors, these patients seem to have more problems compared to the other classes with addiction and violation of agreements related to addiction [15].

## The present study

The goal of the current study was to investigate the predictive validity of the clinical items for violent recidivism, taking into account the heterogeneity of forensic psychiatric patients. Therefore, latent classes of patients were investigated first. Although the clinical items correlate less strongly with violent recidivism than the historical items [18, 19], their predictive validity is especially of interest for optimizing treatment. Focusing on the clinical domain at item-level can help determine the specific focus of follow-up treatment or make decisions about leave modalities [12]. First, we performed LCA to subdivide the group of forensic patients. Despite the exploratory nature of this study, we expected to replicate the five classes described in Van Nieuwenhuizen et al. [15], as a similar procedure was used. Hence, specific hypotheses were formulated based on the classes of Van Nieuwenhuizen et al. [15] for the clinical items of the HKT-R with predictive validity for violent recidivism (as presented in Table 2).

Regardless of classification, it was hypothesized as a general hypothesis that K01 (problem insight), K03 (addiction), K04 (impulsivity), K05 (antisocial behavior), K06 (hostility), K07 (social skills), and K09 (cooperation with treatment) in particular will be predictive of violent reoffending because they correspond to the clinical items of the HKT-30 that had elevated AUC values in predicting severe recidivism [19]. Moreover, K03 (addiction) and K09 (cooperation with treatment) were also related to more inpatient violence during treatment [20]. Another possible clinical item with high predictive validity in general is K12 (violation of terms), which was not part of the HKT-30, but has similarity with item H02 (violation of terms) in the historical domain. According to Hildebrand et al. [21] this item is one of the best predictors of violent recidivism from the HKT-30 and therefore its clinical variant could also be important in predicting violent recidivism. A final note on gender, the HKT-R is not validated for female offenders [9]. Specific risk factors have been found in female offenders, such as covert/manipulative behavior and low self-esteem [22–24]. Although a separate subgroup of women is not expected in this study based on previous findings by Van Nieuwenhuizen et al. [15], we decided to include women to gain insight into the class distribution.

## Method

### Participants and procedure

The initial sample encompassed all patients with a TBS measure who had been discharged unconditionally between 2004 and 2008 from any of 12 Forensic Psychiatric Centers/Clinics (hereinafter referred to collectively as FPCs) (*N* = 347) in the Netherlands. However, of five male patients (1.4%) the possible reconviction data could not be obtained and 10 male patients (2.9%) passed away within two years after discharge, resulting in a final sample of 332 patients. Of these 332 patients, 305 were male (91.9%) and 27 were female (8.1%). The mean age at admission was 31.86 (*SD* = 8.58; range: 17.40–62.01) and at discharge 40.05 (*SD* = 8.90; range: 24.77–69.47). For all these patients, the HKT-R was scored retrospectively based on information obtained from their TBS records, containing thorough descriptions of the patient’s background and criminal history, psychiatric evaluation reports, treatment plans, leave requests, TBS prolongation advices, court evaluations and probation progress reports. This HKT-R scoring was performed by 20 for the purpose intensively trained graduate psychology students for five timepoints per patient, namely: judicial psychiatric observation/investigation, admission in the FPC, permission for unguided leave, conditional release, and unconditional release. To assess the interrater reliability, 60 randomly selected files were scored twice by two independent raters. Subsequently, a one-way random, single measure intraclass correlation coefficient (ICC) was calculated. The ICC for the rating of the HKT-R in total was considered reasonable to good (ICC = 0.62 [0.41-0.77]), and for the clinical domain in particular very good (ICC = 0.85 [0.67-0.94]) [25]. Moreover, this exact data was used in previous studies to respectively investigate the predictive validity of the HKT-R and network configuration of the clinical items [11, 13]. In this study, only two of the five documented time points were used: admission in the FPCs and unconditional release. The data collection was approved by the Scientific Research Committee of FPC Kijvelanden. Moreover, permission was granted by the Dutch Ministry of Justice and Security and the boards of the 12 FPCs included. The data was extended anonymously and could not be traced to individual patients.

### Outcome measures of reoffending

Outcome measures were compiled using a database containing official recidivism data provided by The Ministry of Justice and Security. Each patient discharged between 2004 and 2008 was followed from the time of discharge until five years later. This resulted in two outcome measures per patient, violent reoffending within two and five years. In this context, violent recidivism contained any new conviction for moderate violence, property crime with violence, serious violence, arson with risk for life, (attempted) homicide/murder, and violent sexual assaults. Importantly, recidivism here was measured as receiving a new conviction, meaning that absence of a new conviction did not automatically imply absolute absence of recidivism.

### Instrument

The HKT-R is an SPJ risk assessment instrument consisting of 33 items divided into three domains (Historical, Clinical and Future domain, see Table 1), of which it is assumed that each item is correlated with recidivism upon release [9]. Therefore, these factors are extrapolated and weighed for the likelihood of violent reoffending in forensic psychiatric patients. The historical domain contains 12 items related to the patients’ background and criminal history up until the index offense (for which the TBS measure was imposed), hence exclusively static risk factors. The clinical domain consists of 14 dynamic risk factors and is assessed over the behavior of the past 12 months within treatment. Finally, the seven items of the future domain are used to estimate potential risks with respect to the newly learned skills in treatment, for leave modalities or discharge [9]. All items are scored on a 5-point scale, with a range from 0 (*no risk*) to 4 (*high risk*). Together, this results in an actuarial total score between 0 and 132. Ultimately, a professional clinical judgment is given, manifested in low, low/medium, medium, medium/high, or high risk. Consequently, the total score and professional judgement are combined resulting in a weighted structured clinical final judgment [9].

### Psychopathology

Psychopathology was indicated by diagnoses on Axis I and Axis II of the Diagnostic and Statistical Manual of Mental Disorders (4th ed. text rev.; DSM-IV-TR; [26]), which was the most recent version at that time. These diagnoses were gathered from the patient files and categorized into seven dummy variables for Axis I: no diagnosis, mood disorder, anxiety disorder, developmental disorder (e.g., attention deficit hyperactivity disorder or autistic disorder), psychotic disorder, sexual disorder, substance use disorder (SUD), and other. Dummies were chosen to allow comorbidity among diagnoses on Axis I. As for Axis II, informing about possible personality disorders (PD), five categories were formed within one variable (0 *= no PD*, 1 *= PD cluster A*, 2 *= PD cluster B*, 3 *= PD cluster C*, 4 *= PD NOS [not otherwise specified]*, and 5 *= mix of PDs*).

### Criminal history

Previous convictions were obtained from the patient files and subdivided into 12 categories following the BOOG-systematics [27, 28]. For each category it was counted how many crimes were committed based on the patient file. These categories were operationalized in six ordinal variables. First, ‘nonviolent offenses’ containing the number of offenses from BOOG 1 (*traffic violation and disruption of order*), 2 (*drug-related offenses*), 3 (*destruction of property*) to 4 (*capital and profit*). The second option was ‘light/medium violent offenses’, encompassing all offenses classified in BOOG 5 (*moderate violence and possession of weapons*) and 6 (*property crime with violence*). Thirdly, ‘severe violent offenses’ were captured by the eponymous BOOG 7 (*severe violence*). The fourth, ‘sexual offenses’ contained both offenses within BOOG 8 (*sexual assaults*) as 9 (*sexual assaults with an underaged victim*). Fifth, the number of offenses concerning ‘arson’ were indicated by BOOG 11 (*arson*). Lastly, the sixth category encompassed offenses as well within BOOG 10 (*homicide*) as 12 (*murder*). Although scores on these separate BOOG categories could range from 0 to even 178 offenses within a patient, the scores on these six variables were adjusted so that they could range from 0 to 10 offenses per category, making analyses more manageable. This means that a score of 10 could be 10 offenses or more.

### Statistical analyses

Descriptive statistics were analysed in SPSS version 26 and missing values of the HKT-R were imputed using the Expectation-Maximization algorithm [29]. In total there were 116/3984 missing values on the items of the historical domain, 942/4648 on the clinical domain, and 153/2324 on the future domain. These missing values are mostly attributed to (partly) missing files. Based on Little’s MCAR test it appears that the missing values are missing at random (${\chi }^{2}$(5587) = 5692.93, *p* = .158). Subsequently, the data was transferred into Latent GOLD version 5.1 to identify classes by an explorative LCA. LCA is a model-based probabilistic cluster technique to predict classes based on indicators. Latent GOLD can estimate multiple models simultaneously to select the best fitting model [16]. In the current study we used the three-step approach [30], meaning that in the first step the best fitting LCA model was chosen based on the indicator variables psychopathology and criminal history. To establish the number of classes, the Bayesian information criterion (BIC) and Akaike information criterion (AIC) were used, where lower values meant a better fit of the model [31]. In the second step, participants were assigned to the latent clusters based on probabilities using the classification type “proportional” and maximum likelihood bias-adjustment type. Entropy R^2^ was used to evaluate the quality of the determined classification, where values closer to one indicated a better predictive model [31]. After determining the number of classes, in the third step the resulting classes were compared on external variables, such as the HKT-R items (measured at admission) which were included as dependent covariates (in Latent GOLD). Moreover, the resulting classes were loaded back into SPSS to make comparisons on gender, age, country of birth, intelligence, and duration of treatment, using one-way between subject ANOVAs and Tukey HSD post hoc tests. Then, the predictive validity of the clinical items was assessed for each class separately. Predictive validity is the core psychometric indicator to express the performance of a risk assessment instrument [32] to discriminate between recidivists and non-recidivists. This can be done by applying the area under the curve (AUC), which is operationalized as: “the probability that a randomly selected individual who engaged in an antisocial act received a higher risk classification than a randomly selected individual who did not” [33]. The AUC value can be retrieved from receiver operating characteristics (ROC) with a range between 0 and 1, where a value of 0.5 is similar to chance and a value of 1 means perfect discrimination between recidivism and non-recidivism [33]. Intermediary AUC values are to be interpreted conservatively [34, 35]: accuracy below 0.60 is low, between 0.60 and 0.70 is marginal, between 0.70 and 0.80 is modest, between 0.80 and 0.90 is moderate, and over 0.90 is high.

## Results

### Latent class analysis

#### Model estimation

First, we estimated models ranging from one- to five-class solutions. Based on the BIC value, which is considered the most reliable measure for class estimation [31], the four-class model was selected as shown in Table 3. After bootstrapping, the four-class model showed a good model fit (*p* = .09). Given the Entropy *R*² value and estimated proportional classification errors, the model has adequate classification quality.

**Table 3:** Classification statistics

No. of classes	L²	BIC	AIC	*p* ^a^	Npar	Class error	Entropy of R²
1	3846,1699	7966,6108	7745,9129	0.16	58	0.00	1
2	3509,4864	7746,0300	7449,2294	0.16	78	0.00	0.998
3	3374,4399	7727,0862	7354,1829	0.15	98	0.05	0.898
**4**	**3255,7435**	**7724,4925**	**7275,4866**	**0.09**	**118**	**0.04**	**0.922**
5	3198,4046	7783,2562	7258,1476	0.10	138	0.08	0.882

*Note.* BIC = Bayesian information criterion; AIC = Akaike information criterion; Npar = number of parameters. The best fitting model is presented in bold

^a^*p* after bootstrapping, indicating model fit when non-significant ($\ge$ 0.05)

#### Description of latent classes

**Fig. 1:**
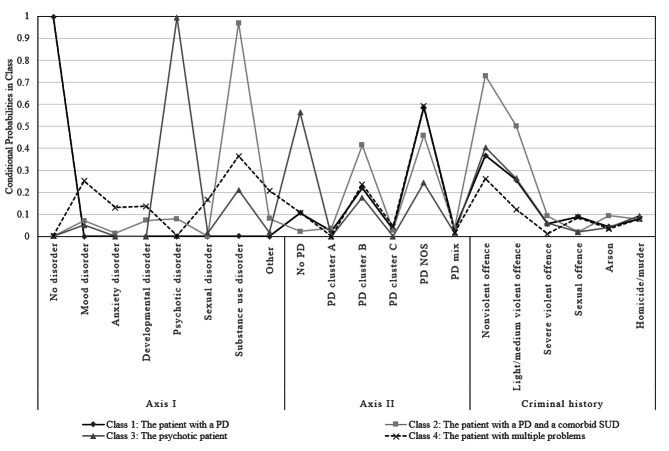
Graphical representation of conditional probabilities by class

The resulting latent classes are shown in Table 4; Fig. 1. The first class, labelled as *The patient with a PD*, comprised 39.4% of the patients (*n* = 131). Compared to the other classes, this class is characterized by patients without an Axis I diagnosis. As for Axis II, patients are most likely to have a PD, but it is not clear whether this is a cluster A, B, C, NOS, or mixed diagnosis, with PD NOS being dominant. The criminal history in the first class is comparable with other classes, with offenses mainly in category 1 (*non-violent*) and 2 (*light/medium violence)*. For offenses in category 4 (*sexual offenses)*, patients in this class did commit significantly more offenses than those in the second and third classes. The second class *The patient with a PD and comorbid SUD* included 23.8% of the patients (*n* = 79). Patients in this class had the highest probability to have a SUD on Axis I, relative to the other classes. Compared to the other classes, patients in this class were significantly more likely to have a cluster B PD and a NOS diagnosis. In addition, compared to the other classes, this class was characterized by significantly more offenses in categories 1 (*non-violent*) and 2 (*light/medium violence).* Next, 18.4% of the patients (*n* = 61) clustered together in the third class *The psychotic patient*. This class consisted of patients with a high probability of a psychotic disorder on Axis I. In addition, they often had a SUD, although significantly less than in the second class. On Axis II, patients had significantly fewer PDs compared to the other classes. Yet, some patients had a cluster B or PD NOS diagnosis. The criminal history was comparable with the first class, with mainly category 1 (*non-violent*) and 2 (*light/medium violence)* offenses. Finally, the fourth class *The patient with multiple problems* contained 18.4% of patients (*n* = 61). Patients in this class were characterized by significantly more mood-, anxiety-, sexual-, and/or other disorders on Axis I than patients in the third class. Moreover, this class was predominantly characterized on Axis II by a PD NOS or cluster B diagnosis. The criminal history in this class was significantly lower than the other classes for category 1 (*non-violent*), 2 (*light/medium violence)*, and 3 (*severe violence)*. In addition, patients had committed significantly more offenses in category 4 (*sexual offenses*) compared to the third class.

**Table 4:** Class-specific probabilities/means of the psychopathology and criminal history

	Class 1 (39.4%)	Class 2 (23.8%)	Class 3 (18.4%)	Class 4 (18.4%)
**Nominal Indicators**	*P (SE)*	*P (SE)*	*P (SE)*	*P (SE)*
*Axis I diagnosis*				
No diagnosis	0.99 (0.00)	0.00 (0.00)	0.00 (0.01)	0.00 (0.01)
Mood disorder	0.00 (0.00)	0.07 (0.03)	0.05 (0.03)	0.25 (0.06)
Anxiety disorder	0.00 (0.00)	0.01 (0.02)	0.00 (0.00)	0.13 (0.05)
Developmental disorder	0.00 (0.00)	0.07 (0.03)	0.00 (0.01)	0.14 (0.05)
Psychotic disorder	0.00 (0.00)	0.08 (0.04)	0.99 (0.01)	0.00 (0.01)
Sexual disorder	0.00 (0.00)	0.00 (0.00)	0.02 (0.02)	0.16 (0.05)
Substance use disorder	0.00 (0.00)	0.97 (0.03)	0.21 (0.06)	0.36 (0.09)
Other	0.00 (0.00)	0.08 (0.03)	0.02 (0.02)	0.21 (0.06)
*Axis II diagnosis*				
No PD	0.11 (0.03)	0.02 (0.02)	0.56 (0.07)	0.11 (0.04)
PD cluster A	0.02 (0.01)	0.04 (0.02)	0.00 (0.00)	0.00 (0.00)
PD cluster B	0.22 (0.04)	0.41 (0.06)	0.18 (0.05)	0.24 (0.06)
PD cluster C	0.03 (0.02)	0.03 (0.02)	0.00 (0.00)	0.05 (0.03)
PD NOS	0.59 (0.04)	0.46 (0.06)	0.24 (0.06)	0.59 (0.07)
Mix of PDs	0.03 (0.02)	0.04 (0.02)	0.02 (0.02)	0.02 (0.02)
**Ordinal Indicator**	*M (SE)*	*M (SE)*	*M (SE)*	*M (SE)*
*Criminal history*				
Non-violent offense	3.67 (0.33)	7.30 (0.51)	4.05 (0.50)	2.63 (0.50)
Light/medium violent offense	2.57 (0.25)	5.02 (0.52)	2.63 (0.40)	1.22 (0.28)
Severe violent offense	0.40 (0.07)	0.65 (0.14)	0.42 (0.11)	0.08 (0.04)
Sexual offense	0.89 (0.19)	0.20 (0.10)	0.20 (0.09)	0.86 (0.28)
Arson	0.18 (0.05)	0.38 (0.10)	0.16 (0.06)	0.14 (0.06)
Murder/homicide	0.73 (0.08)	0.69 (0.11)	0.85 (0.15)	0.72 (0.13)

*Note*. For the nominal indicators (dichotomous) the conditional probability (*P*) is given, this represents posterior class membership probability. For the ordinal indicator the mean number of offenses per class is given (*M*), which could range from 0 to 10 offenses. For both, the standard error is presented between brackets

#### External variables

**Fig. 2:**
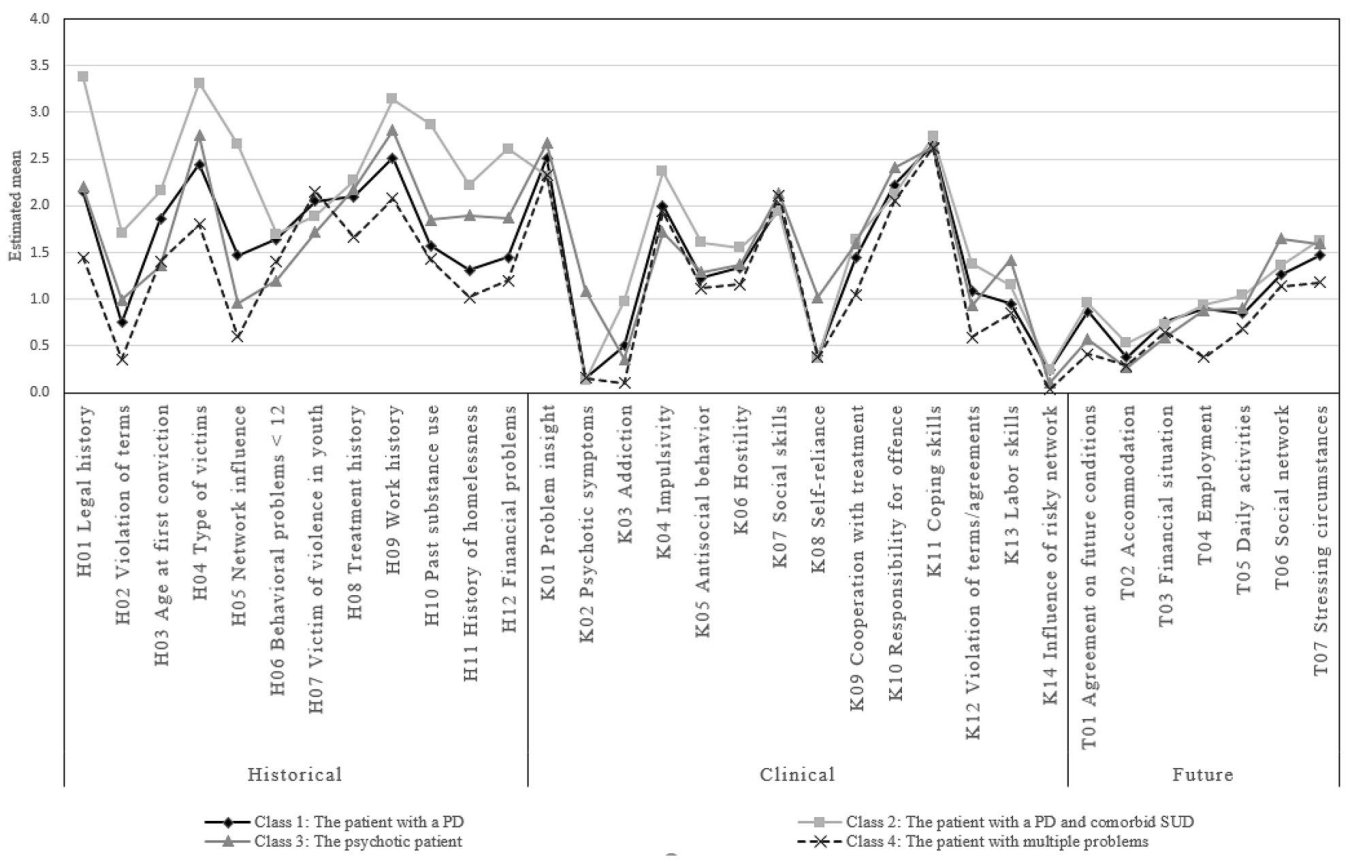
Graphical representation of estimated means on the items of the HKT-R per class

In the next step, all 33 HKT-R items were added to the classes as external variables (see Table 5; Fig. 2). These were the 12 historical items, 14 clinical items at time of admission in the FPC, and the seven future items at unconditional discharge. For the first class *The patient with a PD*, the mean scores were average compared to the other classes. The second class *The patient with a PD and comorbid SUD* showed generally higher mean scores on the historical items compared to the other classes. More specifically, the mean scores on items H01 (legal history), H02 (violation of terms), H04 (type of victims), H05 (network influence), H10 (past substance use), and H12 (financial problems) were significantly higher than in all other classes. As for the clinical and future items, the mean scores were comparable with the other classes, although the mean score on K03 (addiction) was significantly higher. The third class *The psychotic patient*, generally showed little differences with the other classes. Yet, the mean scores on K02 (psychotic symptoms) and K08 (self-reliance) were significantly higher than those in the other classes. Finally, the mean scores in the fourth class *The patient with multiple problems* were relatively low on all items compared to the other classes. Mean scores on H01 (legal history) and H04 (type of victims) in particular were significantly lower than in all other classes.

**Table 5:** Class-specific means of the HKT-R items

	Class 1	Class 2	Class 3	Class 4	Wald
	*M (SE)*	*M (SE)*	*M (SE)*	*M (SE)*	
H01 Legal history	2.15 (0.12)	3.38 (0.10)	2.20 (0.21)	1.45 (0.16)	63.84***
H02 Violation of terms	0.75 (0.10)	1.71 (0.16)	0.99 (0.15)	0.36 (0.13)	26.70***
H03 Age at first conviction	1.86 (0.10)	2.16 (0.15)	1.36 (0.12)	1.41 (0.22)	13.65***
H04 Type of victims	2.44 (0.11)	3.31 (0.11)	2.75 (0.16)	1.80 (0.20)	32.29***
H05 Network influence	1.47 (0.14)	2.66 (0.17)	0.96 (0.18)	0.60 (0.17)	43.91***
H06 Behavioral problems < 12	1.64 (0.13)	1.70 (0.16)	1.20 (0.17)	1.40 (0.18)	5.29
H07 Victim of violence in youth	2.05 (0.11)	1.89 (0.14)	1.72 (0.14)	2.15 (0.17)	5.13
H08 Treatment history	2.10 (0.14)	2.27 (0.13)	2.18 (0.16)	1.66 (0.19)	6.44
H09 Work history	2.51 (0.11)	3.14 (0.13)	2.81 (0.13)	2.08 (0.21)	15.93*
H10 Past substance use	1.57 (0.09)	2.87 (0.09)	1.85 (0.14)	1.43 (0.15)	72.61***
H11 History of homelessness	1.31 (0.11)	2.22 (0.17)	1.90 (0.20)	1.02 (0.18)	27.61***
H12 Financial problems	1.45 (0.12)	2.61 (0.15)	1.87 (0.18)	1.20 (0.18)	34.41***
K01 Problem insight	2.51 (0.07)	2.32 (0.13)	2.67 (0.14)	2.33 (0.13)	4.93
K02 Psychotic symptoms	0.15 (0.04)	0.13 (0.05)	1.09 (0.12)	0.16 (0.08)	32.22***
K03 Addiction	0.50 (0.09)	0.98 (0.15)	0.36 (0.10)	0.10 (0.07)	17.31***
K04 Impulsivity	2.00 (0.11)	2.37 (0.14)	1.72 (0.15)	1.94 (0.19)	9.36*
K05 Antisocial behavior	1.23 (0.12)	1.61 (0.17)	1.29 (0.16)	1.12 (0.18)	4.16
K06 Hostility	1.34 (0.09)	1.55 (0.12)	1.37 (0.14)	1.16 (0.15)	3.39
K07 Social skills	1.99 (0.08)	1.94 (0.12)	2.13 (0.10)	2.11 (0.12)	2.01
K08 Self-reliance	0.39 (0.07)	0.37 (0.10)	1.02 (0.14)	0.38 (0.10)	23.45***
K09 Cooperation with treatment	1.44 (0.10)	1.64 (0.15)	1.59 (0.13)	1.05 (0.19)	5.15
K10 Responsibility for offense	2.22 (0.09)	2.12 (0.13)	2.41 (0.10)	2.05 (0.14)	5.48
K11 Coping skills	2.66 (0.08)	2.74 (0.10)	2.63 (0.11)	2.62 (0.12)	0.74
K12 Violation of terms/agreements	1.08 (0.11)	1.38 (0.18)	0.93 (0.15)	0.59 (0.19)	5.6
K13 Labor skills	0.95 (0.10)	1.15 (0.15)	1.42 (0.16)	0.85 (0.21)	7.60
K14 Influence of network	0.24 (0.05)	0.25 (0.09)	0.10 (0.04)	0.03 (0.03)	7.26
T01 Agreement on future conditions	0.87 (0.10)	0.96 (0.13)	0.57 (0.11)	0.41 (0.10)	11.97**
T02 Accommodation	0.38 (0.07)	0.53 (0.11)	0.27 (0.08)	0.29 (0.09)	4.47
T03 Financial situation	0.76 (0.10)	0.73 (0.11)	0.59 (0.12)	0.65 (0.15)	1.29
T04 Employment	0.90 (0.11)	0.94 (0.15)	0.88 (0.16)	0.38 (0.15)	4.31
T05 Daily activities	0.85 (0.10)	1.04 (0.13)	0.90 (0.14)	0.68 (0.14)	2.84
T06 Social network	1.26 (0.09)	1.36 (0.12)	1.65 (0.14)	1.14 (0.15)	7.50
T07 Stressing circumstances	1.47 (0.09)	1.63 (0.12)	1.59 (0.09)	1.18 (0.14)	5.98

*Note.* Estimated means on the HKT-R items per class. Wald test indicated the associations between class membership and the item. Significance is indicated by *p < .05, **p < .01, and ***p < .001

### Group description

There were no significant differences between the four classes for gender, but country of birth did differ significantly (see Table 6). The proportion of patients born in the Netherlands is highest in classes 2 and 4, and lowest in class 3. Following the results of the ANOVA, there was a significant effect of class membership on age at admission [*F*(3, 328) = 5.434, *p* = .001] and at discharge [*F*(3, 328) = 3.872, *p* = .010]. Post hoc comparisons with the Tukey HSD test indicated that age statistically differed for the first and second class both at admission (∆*M* = -4.57, *SD* = 1.20, *p* < .001) and discharge (∆*M* = -3.45, *SD* = 1.25, *p* = .031), meaning that patients in the second class were significantly older than patients in the first class at admission and discharge of the FPCs. Age at discharge also statistically differed for the first and third class (∆*M* = 3.68, *SD* = 1.36, *p* = .037), in the sense that patients in the third class were significantly older than patients in the first class at discharge from the FPCs. Similarly, for duration of treatment, there was a significant effect of class membership [*F*(3, 328) = 4.098, *p* = .007]. Post hoc comparisons (Tukey HSD) showed a significant difference in duration of treatment for the second and third classes (∆*M =* -1.86, *SD* = 0.55, *p* = .004), meaning that patients in the third class had a significantly longer duration of treatment than patients in the second class. There were no significant differences on mean IQ scores between the classes [*F*(3, 313) = 0.409, *p* = .747].

**Table 6:** Group description per class

		Class 1 (*n* = 131)	Class 2 (*n* = 79)	Class 3 (*n* = 61)	Class 4 (*n* = 61)	Group differences
Gender (%)	Male	120	76	57	52	${\chi }^{2}$ = 5.782
	Female	11	3	4	9	
Country of birth (%)	The Netherlands	73.3%	87.3%	60.7%	85.2%	${\chi }^{2}$ = 22.515**
	Suriname	9.2%	7.6%	18.0%	1.6%	
	The Netherlands Antilles	6.2%	3.8%	8.2%	3.3%	
	Other	11.3%	1.3%	13.1%	9.9%	
Mean age at admission in years (*SD*)		29.68 (8.37)	34.25 (8.94)	32.61 (6.62)	32.71 (9.37)	*F*(3, 328) = 5.434**
Mean age at discharge in years (*SD*)		38.04 (8.33)	41.49 (9.35)	41.72 (7.30)	40.81 (10.26)	*F*(3, 328) = 3.868*
Mean duration of treatment in years (*SD*)		8.37 (3.34)	7.24 (2.66)	9.11 (3.58)	8.10 (3.21)	*F*(3, 328) = 4.098**
Mean IQ (*SD*)		98.24 (14.59)	99.10 (13.95)	96.33 (18.15)	99.23 (18.02)	*F*(3, 328) = 0.409

*Note.* Significant group differences are indicated by *p < .05, **p < .01, and ***p < .001

### Prevalence of reoffending for two- and five-year time at risk

For the total sample, 332 patients were located at the follow-up measurements two years after discharge, of which 41 patients (12.3%) had violently reoffended ($\ge$ 1 offense). For the first class *The patient with a PD*, this meant that 11 (8.4%) of 131 patients had reoffended in a violent offense. For the second class *The patient with a PD and comorbid SUD*, 22 (27.8%) of the 79 patients reoffended. For the third class *The psychotic patient*, three (4.9%) of 61 patients reoffended and five (8.2%) of 61 patients reoffended for the fourth class *The patient with multiple problems*.

At follow-up after five years, only 187 patients could be traced. Reasons for not finding criminal records (*n* = 160) could not be specified precisely for all patients by the Dutch Ministry of Security and Justice. Some patients moved abroad, were in detention, had no residential address, passed away or criminal files could not be found. Of the 187 patients that could be traced, 36 (19.3%) had reoffended with a violent offense ($\ge$ 1). After clustering, this meant that in the first class *The patient with a PD*, 15 (20.0%) of 70 patients violently reoffended. In the second class *The patient with a PD and comorbid SUD*, 13 (30.2%) of 43 patients reoffended. In the third class *The psychotic patient*, five (13.9%) of 36 patients reoffended and in the fourth class, *The patient with multiple problems*, three (9.1%) of 33 patients reoffended.

### Predictive validity

#### Violent recidivism within two years at admission and discharge

Both at admission in the FPC and discharge, the total clinical domain showed low to marginal accuracy in predicting violent reoffending for two-year time at risk for the four classes (AUC = 0.53-0.67, see Table 7). However, when looking at item-level, it was found that for the first class *The patient with a PD* at admission, items K01 (problem insight), K04 (impulsivity), K05 (antisocial behavior), K09 (cooperation with treatment), K10 (responsibility for offense), and K13 (labor skills) were marginal predictors of violent reoffending (AUC = 0.61-0.68). At discharge, K03 (addiction), K04 (impulsivity), K05 (antisocial behavior), K06 (hostility), K09 (cooperation with treatment), K10 (responsibility for offense), K11 (coping), and K12 (violation of terms) marginally predicted violent recidivism (AUC = 0.60-0.69). Meanwhile, K13 (labor skills; AUC = 0.73) was found to be a modest predictor at discharge. For the second class *The patient with a PD and comorbid SUD*, at admission, items K03 (addiction; AUC = 0.67) and K12 (violation of terms; AUC = 61) were found to be marginal predictors. Furthermore, at discharge, K09 (cooperation with treatment; AUC = 0.62) and K13 (labor skills; AUC = 0.62) marginally predicted violent reoffending within two years. Next, for the third class *The psychotic patient*, at admission, the items K10 (responsibility for offense), K13 (labor skills), and K14 (influence of network) were found to be marginal predictors (AUC = 0.62-0.64). Items K03 (addiction), K04 (impulsivity), and K11 (coping skills) were modestly predictive of violent recidivism (AUC = 0.72-0.75). Likewise, at discharge, items K06 (hostility), K08 (self-reliance), K11 (coping skills), and K13 (labor skills) showed to be marginal predictors (AUC = 0.60-0.66). Meanwhile, items K04 (impulsivity), K05 (antisocial behavior), and K07 (social skills) even showed modest accuracy in predicting violent recidivism (AUC = 0.71-0.79). Finally, for the fourth class *The patient with multiple problems*, at admission, the item K02 (psychotic symptoms; AUC = 0.66) showed to be a marginal predictor. Items K03 (addiction; AUC = 0.78) and K07 (social skills; AUC = 0.70) even modestly predicted violent reoffending. Moreover, K06 (hostility; AUC = 0.80) at admission was found to be a moderate predictor. At discharge, K02 (psychotic symptoms), K06 (hostility), K09 (cooperation with treatment), and K12 (violation of terms) marginally predicted violent reoffending in the fourth class (AUC = 0.62-0.68).

**Table 7:** Predictive validity of the Clinical items per class for two year Violent Recidivism

	Class 1		Class 2		Class 3		Class 4	
	AUC *(SE)*	95% CI	AUC *(SE)*	95% CI	AUC *(SE)*	95% CI	AUC *(SE)*	95% CI
*Admission*								
K01	**0.62 (0.09)**	[0.44, 0.79]	0.49 (0.08)	[0.34, 0.63]	0.58 (0.08)	[0.43, 0.73]	0.46 (0.13)	[0.21, 0.71]
K02	0.49 (0.09)	[0.31, 0.67]	0.47 (0.07)	[0.33, 0.62]	0.59 (0.19)	[0.22, 0.96]	**0.66 (0.15)**	[0.36, 0.95]
K03	0.58 (0.10)	[0.38, 0.77]	**0.67 (0.07)**	[0.54, 0.81]	**0.72 (0.16)**	[0.41, 1.0]	**0.78 (0.14)**	[0.51, 1.0]
K04	**0.65 (0.09)**	[0.47, 0.83]	0.47 (0.07)	[0.33, 0.61]	**0.75 (0.10)**	[0.55, 0.95]	0.55 (0.15)	[0.25, 0.85]
K05	**0.62 (0.10)**	[0.43, 0.82]	0.56 (0.07)	[0.42, 0.70]	0.51 (0.18)	[0.17, 0.85]	0.53 (0.15)	[0.24, 0.81]
K06	0.59 (0.10)	[0.40, 0.77]	0.58 (0.07)	[0.44, 0.71]	0.41 (0.08)	[0.25, 0.56]	**0.80 (0.15)**	[0.50, 1.0]
K07	0.53 (0.09)	[0.36, 0.70]	0.54 (0.07)	[0.40, 0.68]	0.59 (0.13)	[0.33, 0.84]	**0.70 (0.10)**	[0.50, 0.90]
K08	0.51 (0.09)	[0.33, 0.69]	0.52 (0.08)	[0.37, 0.67]	0.51 (0.14)	[0.23, 0.80]	0.49 (0.14)	[0.22, 0.76]
K09	**0.60 (0.09)**	[0.44, 0.77]	0.53 (0.07)	[0.39, 0.66]	0.54 (0.21)	[0.14, 0.95]	0.52 (0.14)	[0.25, 0.80]
K10	**0.61 (0.09)**	[0.44, 0.78]	0.50 (0.07)	[0.36, 0.65]	**0.68 (0.17)**	[0.36, 1.0]	0.42 (0.12)	[0.19, 0.65]
K11	0.52 (0.11)	[0.31, 0.74]	0.42 (0.07)	[0.28, 0.56]	**0.75 (0.11)**	[0.54, 0.95]	0.55 (0.13)	[0.30, 0.80]
K12	0.54 (0.11)	[0.33, 0.75]	**0.61 (0.07)**	[0.47, 0.76]	0.34 (0.13)	[0.10, 0.59]	0.57 (0.15)	[0.28, 0.86]
K13	**0.63 (0.09)**	[0.46, 0.80]	0.58 (0.07)	[0.44, 0.71]	**0.62 (0.21)**	[0.22, 1.0]	0.40 (0.13)	[0.14, 0.65]
K14	0.56 (0.10)	[0.37, 0.76]	0.56 (0.08)	[0.41, 0.70]	**0.62 (0.19)**	[0.26, 0.99]	0.58 (0.15)	[0.29, 0.87]
K total	**0.64 (0.10)**	[0.43, 0.84]	0.56 (0.08)	[0.41, 0.71]	**0.64 (0.16)**	[0.32, 0.96]	**0.64 (0.13)**	[0.39, 0.89]
*Discharge*								
K01	**0.66 (0.09)**	[0.49, 0.83]	0.55 (0.08)	[0.40, 0.70]	0.58 (0.15)	[0.30, 0.87]	0.40 (0.16)	[0.09, 0.70]
K02	0.47 (0.09)	[0.30, 0.64]	0.47 (0.07)	[0.33, 0.61]	0.50 (0.21)	[0.08, 0.92]	**0.68 (0.15)**	[0.39, 0.97]
K03	0.51 (0.09)	[0.33, 0.69]	0.54 (0.08)	[0.39, 0.69]	0.58 (0.17)	[0.24, 0.91]	0.55 (0.14)	[0.27, 0.82]
K04	**0.69 (0.09)**	[0.52, 0.87]	0.55 (0.08)	[0.41, 0.70]	**0.71 (0.20)**	[0.32, 1.0]	0.45 (0.15)	[0.16, 0.74]
K05	**0.60 (0.10)**	[0.40, 0.80]	0.53 (0.08)	[0.38, 0.69]	**0.71 (0.17)**	[0.38, 1.0]	0.49 (0.14)	[0.22, 0.76]
K06	**0.62 (0.10)**	[0.43, 0.82]	0.56 (0.08)	[0.42, 0.71]	**0.66 (0.15)**	[0.36, 0.97]	**0.62 (0.15)**	[0.32, 0.92]
K07	0.53 (0.10)	[0.34, 0.73]	0.55 (0.08)	[0.40, 0.70]	**0.79 (0.07)**	[0.66, 0.93]	0.56 (0.15)	[0.25, 0.86]
K08	0.52 (0.09)	[0.34, 0.70]	0.59 (0.08)	[0.44, 0.74]	**0.60 (0.17)**	[0.27, 0.92]	0.53 (0.14)	[0.26, 0.80]
K09	**0.62 (0.10)**	[0.43, 0.81]	**0.62 (0.07)**	[0.47, 0.77]	0.51 (0.19)	[0.15, 0.88]	**0.65 (0.13)**	[0.39, 0.91]
K10	**0.68 (0.09)**	[0.50, 0.86]	0.44 (0.08)	[0.29, 0.58]	0.59 (0.13)	[0.33, 0.85]	0.51 (0.12)	[0.27, 0.74]
K11	**0.60 (0.11)**	[0.39, 0.81]	0.54 (0.08)	[0.38, 0.70]	**0.66 (0.15)**	[0.36, 0.97]	0.43 (0.15)	[0.14, 0.72]
K12	**0.65 (0.10)**	[0.46, 0.84]	0.55 (0.08)	[0.40, 0.70]	0.44 (0.15)	[0.14, 0.74]	**0.63 (0.15)**	[0.34, 0.91]
K13	**0.73 (0.09)**	[0.55, 0.90]	**0.62 (0.08)**	[0.48, 0.77]	**0.65 (0.18)**	[0.30, 1.0]	0.56 (0.14)	[0.27, 0.84]
K14	0.50 (0.09)	[0.32, 0.68]	0.51 (0.07)	[0.37, 0.66]	0.49 (0.17)	[0.16, 0.82]	0.49 (0.13)	[0.23, 0.75]
K total	**0.67 (0.09)**	[0.50, 0.85]	0.53 (0.09)	[0.37, 0.70]	**0.67 (0.17)**	[0.34, 0.99]	0.58 (0.14)	[0.31, 0.86]

*Note.* Area under the curve (AUC) values retrieved by receiver operating characteristics (ROC) curve ranging from 0 to 1. Standard error (SE) is depicted between brackets, followed by the 95% confidence interval (CI). Item names/descriptions are given in Table 1. In bold, marginal AUC values are provided, in bold and underscored are values that have modest, moderate or even high accuracy [34, 35]

Items: H01 legal history; H02 violation of terms; H03 age at first conviction; H04 type of victims; H05 network influence; H06 behavioral problems < 12; H07 victim of violence in youth; H08 treatment history; H09 work history; H10 past substance use; H11 history of homelessness; H12 financial problems; K01 problem insight; K02 psychotic symptoms; K03 addiction; K04 impulsivity; K05 antisocial behavior; K06 hostility; K07 social skills; K08 self-reliance; K09 cooperation with treatment; K10 responsibility for offense; K11 coping skills; K12 violation of terms; K13 labor skills; K14 influence of network

#### Violent recidivism within five years at admission and discharge

For the total sample, the total clinical domain at admission ranged from very low to modest accuracy (AUC = 0.37-0.71, see Table 8) in predicting violent reoffending for five-year time at risk. At discharge, the total clinical domain ranged from low to marginal accuracy (AUC = 0.55-0.68, see Table 8). On item-level, it was found for the first class *The patient with a PD* that items K04 (impulsivity), K05 (antisocial behavior), K06 (hostility), K07 (social skills), and K12 (violation of terms) at admission, were marginal predictors (AUC = 0.61-0.68). At discharge, items K03 (addiction), K04 (impulsivity), K05 (antisocial behavior), K06 (hostility), K07 (social skills), K09 (cooperation with treatment), K10 (responsibility for offense), K12 (violation of terms), and K13 (labor skills) marginally predicted violent recidivism (AUC = 0.60-0.68). For the second class *The patient with a PD and comorbid SUD*, at admission, K01 (problem insight), K03 (addiction), K05 (antisocial behavior), K06 (hostility), K07 (social skills), K09 (cooperation with treatment), and K12 (violation of terms) were found to be marginal predictors (AUC = 0.63-0.69). Meanwhile, K13 (labor skills; AUC = 0.72) even showed modest prediction for violent reoffending. Moreover, at discharge, it was found that items K01 (problem insight), K06 (hostility), K09 (cooperation with treatment), K12 (violation of terms), and K13 (labor skills) were marginal predictors (AUC = 0.60-0.66). In the third class *The psychotic patient*, the items K07 (social skills; AUC = 0.62) and K11 (coping skills; AUC = 0.63) showed marginal prediction at admission. Item K03 (addiction; AUC = 0.72) was found to be modestly predictive of violent recidivism. Meanwhile, at discharge, items K03 (addiction), K07 (social skills), and K10 (responsibility for offense) showed to be marginal predictors (AUC = 0.62-0.65). Item K06 (hostility; AUC = 0.71) at discharge was found to be modestly predictive. Lastly, for the fourth class *The patient with multiple problems* at admission, the items K05 (antisocial behavior), K09 (cooperation with treatment), and K14 (influence of network) were marginally predictive (AUC = 0.60-0.64). Moreover, items K07 (social skills; AUC = 0.72) and K12 (violation of terms; AUC = 0.75) were found to be modest predictors. Items K03 (addiction; (AUC = 0.98) and K06 (hostility; AUC = 0.95) can be interpreted as highly predictive of violent reoffending. At discharge, items K03 (addiction), K04 (impulsivity), and K07 (social skills) were marginal predictors (AUC = 0.60-0.68). Meanwhile, items K06 (hostility; AUC = 0.76) and K12 (violation of terms; AUC = 0.78 were modest. Item K09 (cooperation with treatment; AUC = 0.82) was even moderately predictive in the fourth class at discharge.

**Table 8:** Predictive validity of the Clinical items per class for five year Violent Recidivism

	Class 1		Class 2		Class 3		Class 4	
	AUC *(SE)*	95% CI	AUC *(SE)*	95% CI	AUC *(SE)*	95% CI	AUC *(SE)*	95% CI
*Admission*								
K01	0.50 (0.08)	[0.35, 0.65]	**0.63 (0.10)**	[0.44, 0.82]	0.46 (0.13)	[0.20, 0.71]	0.46 (0.17)	[0.12, 0.80]
K02	0.51 (0.09)	[0.35, 0.68]	0.49 (0.10)	[0.30, 0.68]	0.47 (0.15)	[0.19, 0.76]	0.43 (0.16)	[0.12, 0.74]
K03	0.58 (0.09)	[0.41, 0.75]	**0.69 (0.09)**	[0.51, 0.87]	**0.72 (0.14)**	[0.45, 1.0]	**0.98 (0.02)**	[0.94, 1.0]
K04	**0.63 (0.10)**	[0.44, 0.82]	0.57 (0.10)	[0.38, 0.77]	0.56 (0.16)	[0.25, 0.87]	0.57 (0.21)	[0.15, 0.98]
K05	**0.61 (0.09)**	[0.43, 0.78]	**0.65 (0.10)**	[0.46, 0.84]	0.37 (0.16)	[0.07, 0.68]	**0.64 (0.20)**	[0.26, 1.0]
K06	**0.68 (0.08)**	[0.54, 0.83]	**0.66 (0.09)**	[0.49, 0.84]	0.28 (0.10)	[0.08, 0.48]	**0.95 (0.04)**	[0.88, 1.0]
K07	**0.61 (0.08)**	[0.46, 0.76]	**0.64 (0.08)**	[0.48, 0.80]	**0.62 (0.13)**	[0.37, 0.87]	**0.72 (0.12)**	[0.49, 0.94]
K08	0.56 (0.09)	[0.39, 0.72]	0.56 (0.10)	[0.36, 0.75]	0.33 (0.10)	[0.13, 0.54]	0.54 (0.19)	[0.18, 0.91]
K09	0.54 (0.09)	[0.37, 0.71]	**0.63 (0.09)**	[0.46, 0.81]	0.40 (0.15)	[0.11, 0.70]	**0.64 (0.19)**	[0.27, 1.0]
K10	0.53 (0.09)	[0.36, 0.70]	0.52 (0.09)	[0.34, 0.71]	0.54 (0.14)	[0.27, 0.81]	0.40 (0.18)	[0.05, 0.75]
K11	0.51 (0.10)	[0.32, 0.70]	0.51 (0.10)	[0.31, 0.71]	**0.63 (0.14)**	[0.36, 0.90]	**0.60 (0.17)**	[0.26, 0.94]
K12	**0.65 (0.09)**	[0.48, 0.82]	**0.65 (0.10)**	[0.47, 0.84]	0.37 (0.15)	[0.07, 0.67]	**0.75 (0.17)**	[0.41, 1.0]
K13	0.59 (0.08)	[0.44, 0.75]	**0.72 (0.08)**	[0.56, 0.88]	0.38 (0.17)	[0.05, 0.71]	0.46 (0.19)	[0.08, 0.83]
K14	0.49 (0.08)	[0.33, 0.66]	0.48 (0.10)	[0.30, 0.67]	0.55 (0.15)	[0.26, 0.84]	**0.64 (0.20)**	[0.26, 1.0]
K total	**0.66 (0.08)**	[0.51, 0.80]	**0.70 (0.08)**	[0.53, 0.87]	0.37 (0.18)	[0.01, 0.72]	**0.71 (0.17)**	[0.37, 1.0]
*Discharge*								
K01	0.52 (0.09)	[0.34, 0.71]	**0.60 (0.09)**	[0.42, 0.78]	0.33 (0.10)	[0.13, 0.53]	0.29 (0.13)	[0.03, 0.55]
K02	0.47 (0.08)	[0.31, 0.63]	0.48 (0.10)	[0.30, 0.67]	0.44 (0.14)	[0.17, 0.72]	0.48 (0.17)	[0.15, 0.82]
K03	**0.60 (0.09)**	[0.43, 0.78]	0.59 (0.10)	[0.39, 0.79]	**0.64 (0.15)**	[0.35, 0.94]	**0.64 (0.19)**	[0.27, 1.0]
K04	**0.65 (0.08)**	[0.49, 0.80]	0.57 (0.10)	[0.37, 0.76]	0.59 (0.15)	[0.31, 0.88]	**0.60 (0.18)**	[0.24, 0.96]
K05	**0.64 (0.08)**	[0.48, 0.80]	0.59 (0.10)	[0.39, 0.78]	0.45 (0.13)	[0.19, 0.71]	0.56 (0.19)	[0.18, 0.94]
K06	**0.66 (0.08)**	[0.50, 0.82]	**0.60 (0.10)**	[0.41, 0.79]	**0.71 (0.11)**	[0.49, 0.94]	**0.76 (0.18)**	[0.42, 1.0]
K07	**0.62 (0.08)**	[0.45, 0.79]	0.56 (0.10)	[0.36, 0.75]	**0.65 (0.10)**	[0.45, 0.85]	**0.68 (0.19)**	[0.30, 1.0]
K08	0.46 (0.08)	[0.30, 0.62]	0.57 (0.10)	[0.37, 0.77]	0.41 (0.12)	[0.18, 0.64]	0.59 (0.19)	[0.23, 0.96]
K09	**0.63 (0.09)**	[0.46, 0.80]	**0.61 (0.10)**	[0.42, 0.81]	0.39 (0.12)	[0.15, 0.63]	**0.82 (0.07)**	[0.68, 0.97]
K10	**0.60 (0.09)**	[0.43, 0.77]	0.54 (0.10)	[0.34, 0.73]	**0.62 (0.12)**	[0.39, 0.84]	0.40 (0.17)	[0.06, 0.74]
K11	0.54 (0.08)	[0.38, 0.70]	0.45 (0.11)	[0.25, 0.66]	0.50 (0.12)	[0.27, 0.72]	0.54 (0.20)	[0.16, 0.93]
K12	**0.64 (0.09)**	[0.47, 0.81]	**0.61 (0.10)**	[0.41, 0.80]	0.53 (0.15)	[0.24, 0.83]	**0.78 (0.16)**	[0.46, 1.0]
K13	**0.68 (0.08)**	[0.52, 0.83]	**0.66 (0.10)**	[0.47, 0.85]	0.48 (0.13)	[0.22, 0.74]	0.58 (0.19)	[0.21, 0.95]
K14	0.44 (0.08)	[0.29, 0.60]	0.50 (0.10)	[0.31, 0.69]	0.50 (0.14)	[0.22, 0.78]	0.48 (0.17)	[0.15, 0.82]
K total	**0.67 (0.08)**	[0.51, 0.82]	**0.64 (0.10)**	[0.44, 0.83]	0.55 (0.10)	[0.35, 0.74]	**0.68 (0.17)**	[0.35, 1.0]

*Note.* Area under the curve (AUC) values retrieved by receiver operating characteristics (ROC) curve ranging from 0 to 1. Standard error (SE) is depicted between brackets, followed by the 95% confidence interval (CI). Item names/descriptions are given in Table 1. In bold, marginal AUC values are provided, in bold and underscored are values that have modest, moderate or even high accuracy [34, 35]

Items: H01 legal history; H02 violation of terms; H03 age at first conviction; H04 type of victims; H05 network influence; H06 behavioral problems < 12; H07 victim of violence in youth; H08 treatment history; H09 work history; H10 past substance use; H11 history of homelessness; H12 financial problems; K01 problem insight; K02 psychotic symptoms; K03 addiction; K04 impulsivity; K05 antisocial behavior; K06 hostility; K07 social skills; K08 self-reliance; K09 cooperation with treatment; K10 responsibility for offense; K11 coping skills; K12 violation of terms; K13 labor skills; K14 influence of network

## Discussion

The aim of this study was twofold: first, LCA was performed within a heterogeneous group of forensic psychiatric patients whose TBS measure was unconditionally terminated in the period between 2004 and 2008. Second, the predictive validity of the HKT-R was investigated for violent recidivism two and five years after discharge, for each class separately at item-level. Four classes were identified: (1) *The patient with a PD* characterized by patients with a PD (cluster B/NOS), without Axis I comorbidity; (2) *The patient with a PD and comorbid SUD* characterized by patients with PD (cluster B/NOS) and comorbid SUD; (3) *The psychotic patient* characterized by patients with a psychotic disorder and sometimes comorbidities with SUDs or PDs (cluster B/NOS) although less than in the other classes; and (4) *The patient with multiple problems* characterized by patients with mood-, anxiety-, sexual- and/or other disorders on Axis I, and sometimes comorbid PD (cluster B/NOS). Overall, the classes showed small differences in criminal history, with mostly non-violent or light/medium violent prior offenses. However, although the patients in the fourth class *The patient with multiple problems* had fewer previous offenses overall than those in the other classes, they did commit more sexual offenses.

The prevalence rates of reoffending varied among the four classes. *The patient with a PD and comorbid SUD* (class 2) had the highest prevalence of violent reoffending both two and five years after discharge. Compared to *The patient with a PD* (class 1), it is notable that the comorbidity of an SUD increases the risk of violent reoffending, which is consistent with previous research [36], in which was also found that comorbid SUD increased the risk of mortality. Substance use is probably not an individual predictor, but is associated with other risk factors [36]. Moreover, type of substance use could have an effect, which was not considered in this study but was found in a similar study focussed on SUD in forensic patients [37]. For instance, alcohol abuse appears to be more related to violent reoffending and drug abuse to general reoffending [38]. Moreover, it is striking that in the first class *The patient with a PD* the patients at admission and discharge were on average younger than the patients of the second class *The patient with a PD and comorbid SUD*. To our knowledge, there is no research to explain this difference in age. Logically, patients with second-class comorbidity, should be expected to have more severe problems and commit offenses at a younger age, given the increased risk of reoffending [36]. However, comorbidity does not automatically mean severity [39], and therefore this finding raises questions about patients with PD in comparison to patients with PD and SUD.

The average length of stay in the FPC was significantly shorter for the second class *The patient with a PD and comorbid SUD* than for the third class *The psychotic patients*, while the prevalence of violent recidivism was the lowest for the third class. A possible explanation may be that patients in the second class show more socially desirable behavior (faking bad/faking good) [40, 41]. Furthermore, we cannot know whether the reoffending patient simply needed more treatment, or had more persistent problems, possibly reinforced by a problematic network, a decline in substance use, or practical problems (finance/residency). The reason and context in which the offense took place are often unknown, even though these are crucial aspects of the person-situation interaction in crime prevention [42]. Lastly, *The psychotic patient* and *The patient with multiple problems* classes had the lowest prevalence rates for violent recidivism, which is consistent with previous meta-analyses [14, 38, 43]. However, clustering greatly reduced sample sizes, especially after five years. This decline has serious consequences for the prevalence of violent reoffending, and the predictive validity.

For the predictive validity of the clinical items in the classes, there were many differences between and within classes. Within classes, there were differences both for the clinical items measured at admission and discharge, and for predicting violent reoffending at two and five years after discharge. Therefore, the resulting picture consisted of two time points for measuring clinical items and two timepoints for measuring violent reoffending, making it rather complex. For the first class *The patient with a PD*, consistent to our hypotheses, lack of problem insight, impulsivity, antisocial behavior, hostility, limitations in social skills, lack of cooperation with treatment, and violation of terms were marginally predictive at multiple time points [19–21]. In addition, limitations in labor skills were found to be marginally predictive of violent reoffending, specifically when measured at discharge. Of the second-class *The patient with a PD and comorbid SUD* clinical items, few items showed predictive value for violent recidivism after two years, while this class had the highest prevalence of reoffending. After five-year time at risk, there were many items with increased predictive validity, which may be explained by the heterogeneity of this class, due to different PDs and especially the different possible substances [37]. Lack of problem insight, addiction, hostility, lack of cooperation with treatment, violation of terms, and limitations in labor skills were marginal predictors of violent recidivism at multiple time points, partly consistent with our general hypotheses [19–21], but also with the more specific hypotheses (Table 2) regarding the *patient suffering from addiction* [15]. In the third class *The psychotic patient*, (at least) marginally predictive items at multiple timepoints were: addiction, impulsivity, hostility, limitations in social skills, lack of responsibility for the offense, limitations in coping-, and labor skills. This is only partly consistent with our hypotheses, as only impulsivity, limitations in social-, and coping skills were expected to be predictive of violent reoffending [14, 17]. Finally, for the fourth class *The patient with multiple problems*, psychotic symptoms, addiction, hostility, limitations in social skills, lack of cooperation with treatment, and violation of terms were found to be (at least) marginally predictive at multiple time points. This was broadly in line with the general hypotheses [19–21], though not with the specific hypothesis based on *the patient with sexual problems* [15, 17], since only limitations in social skills correspond to our findings. These differences could be explained by the previously mentioned broader range of patients within this class compared with previous studies.

### Strength and limitations of the study

Despite the limitation of many missing values, one of the major strengths of this study is that the sample entails all the patients who were discharged from one of the Dutch FPCs between 2004 and 2008, signifying high ecological validity and a representative sample. Furthermore, the focus on the individual clinical items enriches the research on risk assessment because knowledge about individual items is important for treatment and risk management. However, given the recommended sample size of 500–1000 participants to perform LCA [44], our sample size was quite small (*N* = 332). The resulting classes from LCA are also not fully independent of each other, given the probabilistic estimation technique [16]. Moreover, the retrospective score of the HKT-R on file records is inferior to the use of data scored by professionals based on direct behavioral observations. Likewise, psychopathology was assessed before admission to the FPCs, with many PD NOS diagnoses. This can be diagnosed when a person does not meet the full criteria for a specific PD, but still displays severe characteristics causing distress or impairment [26]. Officially, one could argue about this diagnosis since it can be seen as no PD for the person not meeting full criteria. This can be further assessed within the FPC, which means that the PD NOS could change into a specific PD. Nevertheless, we decided to include PD NOS, because concluding that there would be no PD would conflict with the reason for TBS imposition, namely the presence of psychopathology. Although the DSM-IV-TR Axis II officially also captures mental retardation, these diagnoses were not considered in the LCA, though mean IQ scores did not differ across classes. Moreover, we did not control for medication, while in general more than half of the forensic patients is on medication for psychotic decompensation, mood-/anxiety disorders, impulse control, SUD or a sexual disorder [15].

In addition, the official reconvictions that were retrieved could be incomplete because we only had official reconviction data. Therefore, it would have been better to also include police arrests or even more informal reports of crime related behaviors of the patient. More specifically, it can be informative to compare the index offenses with the offenses during reconviction and consider the context in which crimes occurred. In this way, it can be assessed at an individual level whether the reconviction is comparable to the index crime. Furthermore, the HKT-R is merely validated for male offenders who have committed violent offenses and male patients with psychotic vulnerability, PD, or both. This means that for females and sexual offenders with underaged victims, the HKT-R has not been validated, although females and sex offenders have been included in the research design. However, given the advantage of the complete sample, we must keep this in mind when interpreting the results. From an ethical perspective, the predictive validity can only be measured if patients (violently) reoffend. Therefore, this is something we would rather not encounter in society but is required to assess the psychometrics of the instrument. Consequently, if forensic patients did not reoffend, we cannot assess the predictive validity.

Moreover, the small sample sizes at the cluster-level (hence reduced predictive power) might have resulted in Type-I error and a chance-based elevated AUC value rather than a meaningful predictor variable. A limitation of the use of the AUC value specifically, is that it does not differentiate between false positives and false negatives and the acceptable cut-off is debatable [45]. Overall, it can be argued that the accuracy of predictions about future behavior decreases with time and may even be equivalent to chance after five years [46]. Applied to the context of forensic risk assessment, there can be enormous differences in recidivism rates because of the variation in follow-up periods [47]. Moreover, community treatment programs can be offered after (unconditional) discharge [48], so we cannot rule out that some participants in the current study had access to outpatient healthcare and others did not, which could have affected recidivism. More specifically, although risk assessment tools are the best available practice, they remain generally insufficient [49]. This includes the lack of (external) validation, preferably based on minimally 100 violent reconvictions [50], which was not met in the current study for the total group let alone for the subgroups. In addition, control groups without risk assessment are needed to evaluate the effectiveness of the risk assessment tool [47, 49], which was lacking in the current study. Lastly, risk assessment tools are often at risk of racial or ethnic biases and do not consider related contextual characteristics (e.g., neighborhood deprivation) [50].

### Clinical implications and future research

Due to many individual differences, the identified classes in the current study cannot be automatically translated to clinical practice. Given the insufficient sample sizes in classes 3 (*The psychotic patient*) and 4 (*The patient with multiple problems*), the generalizability of the results and thus the clinical implications are compromised and should therefore be taken with caution. More (similar) research on this topic is recommended before concrete clinical implications can be identified. Given the presence of SUD in three of the four classes, future research should specify the nature of addiction because differences in SUD were found to be associated to the types of recidivism [38], and may be related to psychopathology and type of offense [37]. Likewise, it is also important to specify the type of PD. For instance, cluster B PD (specifically antisocial personality disorder) is most common in forensic patients, while the other clusters are far less represented [51, 52]. Furthermore, depending on psychopathology, there are different forms of treatment (e.g., psychotropic medication in case of psychotic disorders or schema therapy in case of PD). However, no information was available about patients’ specific treatment, making it impossible to investigate what exactly caused a decrease in risk factors or the absence of reoffending. Moreover, given the high rate of medication use identified in earlier research [15], information about medication use post release could also inform about potential factors leading to reoffending. Especially, since violent recidivism rates have been found to be lower for ex-prisoners taking psychotropic medication (vs. periods in which the individuals did not take medication) [53]. Moreover, the third class *The psychotic patient* scored significantly lower on violent recidivism compared to the other classes. This suggests that antipsychotics could have a buffering effect on aggression [54]. Lastly, contrary to expectations, we found the clinical item limitations in labor skills a marginal predictor of violent recidivism for three out of four classes. At first sight, this seems negligible, while it is very important for patients to develop work skills to reintegrate in society [55]. Likewise, as described in a review of occupational therapy within the forensic psychiatric population [56], occupational therapy requires more evidence-based techniques and research. Therefore, this item of the HKT-R deserves more attention in future research and in treatment.

## Conclusion

The current research once more emphasizes the heterogeneity in the forensic psychiatric population. In fact, it suggests there are differences in risk and protective factors in different subgroups of forensic patients. This is, despite the limitations, even reflected in the predictive validity of the clinical items of the HKT-R. Given these individual differences in psychopathology, criminal history, and risk factors, it is essential to be careful when interpreting risk assessment tools for individual patients or patient groups. Therefore, it is recommended to identify or consider subgroups when conducting research within the forensic population.

## Data Availability

The data that support the findings of this study is not publicly available given the sensitive nature of the data since it includes forensic patients, the data can be requested (contact: Iris Frowijn; I.Frowijn@fivoor.nl).

## List of Abbreviations

AIC: Akaike information criterion
AUC: Area Under the Curve
BIC: Bayesian information criterion
BOOG: Beslissingsondersteuning Onderzoek Geestvermogens [Decision Guidance Research Mental Capabilities]
DJI: Dienst Justitiële Inrichtingen
FPC: Forensich Psychiatric Center/Clinic
HCR-20^V3^: Historical Clinical Risk-management-20 Version 3
HKT-30: Historisch Klinisch Toekomst – 30 [Historical Clinical Future – 30]
HKT-R: Historisch Klinisch Toekomst – Revised [Historical Clinical Future – Revised]
ICC: Intraclass correlation coefficient
LCA: Latent Class Analysis
PD: Personality disorder
PD NOS: Personality disorder not otherwise specified
ROC: Receiver operating characteristics
SPJ: Structured Professional Judgment
SUD: Substance use disorder
TBS: Terbeschikkingstelling [Dutch Entrustment Act]
